# Comparison of Muscle, Bone and Fat Indices between Stages of Sarcopenia in Postmenopausal Malaysian Women

**DOI:** 10.21315/mjms2023.30.5.8

**Published:** 2023-10-30

**Authors:** Nurdiana Zainol Abidin

**Affiliations:** School of Biosciences, Faculty of Science and Engineering, University of Nottingham Malaysia, Selangor, Malaysia. Department of Community Health, Advanced Medical and Dental Institute, Universiti Sains Malaysia, Pulau Pinang, Malaysia

**Keywords:** obesity, sarcopenia, osteoporosis, muscle strength, bone obesity

## Abstract

**Background:**

The cumulative health impact of sarcopenia when it overlaps with obesity and osteoporosis is poorly understood. This cross-sectional study compared the muscle, bone and fat indices between stages of sarcopenia and determined the association of sarcopenia stages with adiposity and bone density in Malaysian postmenopausal women.

**Methods:**

One hundred and thirty-six postmenopausal Malaysian women from Semenyih and Kuala Lumpur, Malaysia participated in the study. Muscle mass and body fat percentages (BFP) were assessed using a bioelectrical impedance analyser. Bone density was assessed using quantitative calcaneal ultrasonography. Handgrip strength (HGS) was assessed using a handgrip dynamometer. Gait speed was assessed using the 6-m walk test. The sarcopenia stage was classified into pre-sarcopenia, sarcopenia and severe sarcopenia.

**Results:**

The overall prevalence of participants with various stages of ‘sarcopenia’ was 29.4%. The rates of low bone density were 13.7%, 12.5%, 17.4% and 85.7% in the non-sarcopenic, pre-sarcopenic, sarcopenic and severe sarcopenic groups, respectively (*P* < 0.000). Age, adiposity, muscle mass, gait speed and bone density differed significantly between the reference (non-sarcopenic) and ‘severe sarcopenic’ groups. The ‘sarcopenic’ and ‘severe sarcopenic’ groups had common impairments whereby no significant differences were found in HGS and gait speed between them.

**Conclusion:**

The results showed significant correlations between sarcopenia stages and age, body weight, adiposity and bone density. Individuals with ‘sarcopenia’ had the same level of HGS and gait speed as those with severe forms of the disorder, implying that individuals with sarcopenia and severe sarcopenia were at the same level in terms of strength and endurance.

## Introduction

With the onset of menopause, there is a noticeable acceleration in muscle mass loss (1). Women’s muscle mass declines progressively after 30 years old of age and this reduction accelerates around 50 years old of age, which is normally around the age when women experience menopause (2, 3). Studies have reported that lean body mass (LBM) decreased by 0.5% (mean absolute yearly loss of 0.2 kg) throughout the menopausal transition, while fat mass increased by 1.7% each year (mean absolute gain of 0.45 kg) (4). This makes postmenopausal women vulnerable to sarcopenic obesity, in which an individual has a concurrent presence of low muscle mass and high adiposity.

Sarcopenia is an age-related disorder defined as a loss of muscle mass in combination with muscle strength and function. Progression of the disorder can lead to immobility, falls, disability and even death (5). Postmenopausal women were three times more likely to be diagnosed with sarcopenia than younger women (odds ratio [OR] 2.99; 95% confidence interval [CI]: 1.38, 6.51) (6). Sarcopenia is associated with several factors. For example, the loss of muscle mass is considered to be caused by a combination of low muscle fibres and muscle atrophy (7). Another theory proposes that sarcopenia is caused by the denervation of motor units, which are then re-innervated by slower motor units (7). Currently, the aetiology of sarcopenia and its mechanism are not fully understood. Nevertheless, observational studies have shown that the number of satellite cells involved in muscle regeneration is substantially lower in older individuals, suggesting that they may play a role in the development of sarcopenia (8). Other factors that are associated with the development of sarcopenia include a reduced level of growth hormone (GH), insulin-like growth factor (IGF-1) and androgens, which are related to the development of skeletal muscle (9). Furthermore, studies have also implied the involvement of the renin-angiotensin system in muscle function modulation. This theory suggests that circulating angiotensin II is associated with muscle wasting, reduced IGF-1 levels and insulin resistance and, therefore, could potentially contribute to the development of sarcopenia (9). Chronic inflammation is also reportedly associated with sarcopenia, and observational studies have shown increased levels of pro-inflammatory cytokines, tumour necrosis factor-α and interleukin-6 in the ageing muscle of individuals with sarcopenia (10).

Owing to the complexity of the disease, there is no universally accepted definition of sarcopenia, making diagnosis more challenging. Currently, two different diagnostic methods for sarcopenia are available for the European and Asian populations. The European Working Group on Sarcopenia in Older People (EWGSOP) proposed the European diagnostic method in 2010 (and the revised EWGSOP2 in 2019) for European populations (11), while the Asian Working Group of Sarcopenia (AWGS) proposed the Asian diagnostic method in 2014 (and the revised AWGS2 in 2019) for Asian populations. Both methods use different cut-off values for each parameter based on existing studies in their respective societies (12). For both sets of criteria (AWGS and EWGSOP), the severity of sarcopenia was classified into three groups: i) pre-sarcopenia, ii) sarcopenia and iii) severe sarcopenia. ‘Pre-sarcopenia’ is defined as the presence of low muscle mass only. ‘Sarcopenia’ is diagnosed when there is a presence of low muscle mass and either low muscle strength or low physical performance. ‘Severe sarcopenia’ is defined when low muscle mass, low muscle strength and low physical performance are present simultaneously (12, 13).

Currently, little evidence is available on the multiplicative health impact of sarcopenia in a distinct overlap with obesity and osteoporosis. The term used to describe an individual with concurrent obesity, low bone mass (osteoporosis) and low muscle mass (sarcopenia) is osteosarcopenic obesity (OSO) (14–17). OSO is an age-related condition regarded as the most advanced form of functional impairment involving bone, muscle and fat (18). Other manifestations of this disorder include osteopenic obesity (OO) and sarcopenic obesity (SO), wherein ‘obesity’ is characterised not only by its clinical diagnosis but also by fat infiltration in muscle tissues and its effect on the skeleton. Both conditions (OO and SO) may eventually progress to OSO. A better understanding of the relationship between osteoporosis, sarcopenia and obesity may help develop effective preventive and therapeutic strategies for OSO. In the present study, we compared the muscle, bone and adiposity indices between different stages of sarcopenia in postmenopausal Malaysian women and subsequently determined which component of body composition is affected the most at each sarcopenic stage. These findings could illuminate the associations between the sarcopenia stage, adiposity and bone density. If severe sarcopenia is related to the highest adiposity and lowest bone density, the next step will be to further explore the interrelationship between the three body composition components and determine whether obesity is the culprit of low muscle mass and low bone density.

## Methods

### Selection and Recruitment of Participants

Based on the sample size calculation (cross-sectional) (19), at least 91 participants were required for this study (anticipating a 15% attrition rate). Two hundred postmenopausal Malaysian women were recruited from various locations (such as neighbourhoods, churches and mosques) around the Semenyih and Klang Valley areas of Kuala Lumpur, Malaysia through flyers, phone calls, e-marketing and liaisons. The recruitment period was from April 2017 to June 2018. Ultimately, 136 women (45 years old–88 years old) satisfied the screening criteria and were included in the analysis. A participant was considered ‘postmenopausal’ if they had no menstrual period, bleeding or spotting for 12 months before enrolment. Details about the study, including the aims, procedures, advantages, risks and potential discomforts, were briefed to interested participants before recruitment. The following inclusion criteria were used to screen healthy and interested volunteers for eligibility: i) women, ii) citizens of Malaysia and iii) postmenopausal women. Exclusion criteria included i) inability to stand for height, weight, and gait speed assessments; ii) presence of artificial limbs and/or metal implants; iii) severe cardiac, pulmonary or musculoskeletal disorders; iv) severe cognitive impairment or any disability that makes communication impossible; and v) presence of terminal illness. No medical history was recorded.

### Anthropometric and Body Composition Measurements

#### Height

A portable stadiometer (SECA 217, Vogel & Halke GmbH & Co., Germany) was used to measure the height to the nearest 0.1 cm. Participants were instructed to stand with their shoulders, buttocks and heels against the stadiometer, making a 45° angle with their toe tips, heels touching, head held straight and neck in a natural position.

#### Waist Circumference

A measuring tape (SECA 203, GmbH & Co. kg, Hamburg, Germany) was used to determine waist circumference (WC). WC was measured (in cm) halfway between the last rib and the anterior superior iliac spine, with the individual standing erect.

#### Quantitative Ultrasound (QUS) Bone Assessments

Bone density was measured using a calcaneal ultrasonic bone densitometer (SAHARA® Clinical Bone Sonometer; Hologic Inc., Waltham, MA, USA). The SAHARA® system was used to measure the broadband ultrasonic attenuation (BUA) (dB/MHz), which indicates attenuated sound waves, as well as the speed of sound (SOS) (m/s). The sound attenuation of the heel was evaluated without any bias caused by the transducers and/or transducer pads using a reference medium. The reference medium was the SAHARA^®^ QC Phantom (supplied by the SAHARA^®^ device).

#### Body Composition

Body composition was assessed using a multisegmental bioelectrical impedance analyser (BIA) (InBody 230 Body Composition Analyser, Biospace Co. Ltd., Seoul, Korea). While standing on this equipment, the participants’ weight, body fat percentage (BFP), skeletal muscle mass (SMM), fat-free mass, basal metabolic rate, body mass index (BMI), segmental muscle and fat analysis were automatically computed. InBody 230 is a multisegmental BIA that employs eight electrodes (hand-to-foot) to calculate whole-body and regional body composition. The appendicular skeletal muscle mass (appSMM) was calculated by adding the muscle masses of the four limbs. The appSMM index (appSMMI) was defined as the sum of the muscle masses of the four limbs, adjusted for height in square metres (kg)/height^2^ (12).

#### Muscle Strength

A hand dynamometer (JAMAR Hydraulic Hand Dynamometer® Model PC-5030 J1, Fred Sammons, Inc., Burr Ridge, IL, USA) was used to assess the handgrip strength (HGS) in each hand as a proxy for muscle strength. HGS was measured twice, consecutively, with a 5-s break for each hand, with the highest of the two values used in the study. The second handle was used as the standard position, as suggested by the American Society of Hand Therapists (ASHT). The subject was seated, shoulders adducted and neutrally rotated, elbow flexed at 90°, forearm in neutral, and wrist between 0° and 30° of dorsiflexion, according to the ASHT (20).

### Physical Performance

#### Gait Speed

Gait speed was measured using a 6-m normal walk test. The 6-m track was marked with two cones or pieces of tape and measured with a roll-up, a self-retracting construction measuring tape. For the test duration, the participants walked at their normal pace from one end of the course to the other. The timer was started when the tester/instructor said ‘begin’ and stopped when one of the participants’ feet crossed the 6-m marker. If participants normally used canes or other assistive devices for walking, they were allowed to do so during the test. The cut-off value was 0.8 m/s, according to the AWGS recommendations (21).

### Stages of Sarcopenia

The AWGS 2014 guidelines were used to classify sarcopenia stages (21). Participants were stratified into four groups: i) non-sarcopenia, ii) pre-sarcopenia, iii) sarcopenia and iv) severe sarcopenia. The appSMMI, HGS and gait speed were used as the criteria to define sarcopenia. ‘Pre-sarcopenia’ was defined as low muscle mass accompanied by normal muscle strength and gait speed. ‘Sarcopenia’ was characterised by low muscle mass, in addition to low muscle strength or low gait speed. Finally, ‘severe sarcopenia’ was defined as low muscle mass in addition to low muscle strength and low gait speed.

### Statistical Analysis

IBM SPSS Statistics for Windows (IBM Corp., Armonk, NY, USA; version 24.0) was used to conduct statistical analysis. The study participants’ characteristics were reported as mean and standard deviation (SD). Categorical variables were reported as frequencies and percentages. Correlations and differences between proportions were determined using Pearson’s chi-square test. A comparison of various parameters between the sarcopenic groups was performed using a one-way analysis of variance (ANOVA). When significant differences were found with ANOVA, post-hoc Tukey’s honestly significant difference (HSD) test was applied to correct for multiple comparisons. Eta squared (η^2^) for between-group effect sizes were calculated using SPSS and interpreted as follows: i) small effect (< 0.01), ii) small-to-medium effect (0.01–0.10) and iii) medium-to-large (0.10–0.25) (22). Multinomial logistic regression analysis was used to examine the association between sarcopenia stage and age, BUA level, body weight and BFP. Statistical significance was set at a two-tailed *P*-value of ≤ 0.05.

## Results

After screening, 136 postmenopausal women were included in the analysis. Table 1 shows the differences in characteristics between the reference group (non-sarcopenic) and the various stages of sarcopenia (pre-sarcopenia, sarcopenia and severe sarcopenia). The findings showed that individuals in the severe sarcopenia group were significantly older than those in the reference group (*P* < 0.01; η^2^p (eta-squared) = 0.083) (Table 1). The reference group, on average, had a higher body weight, BMI, WC and BFP than the other groups (pre-sarcopenia, sarcopenia and severe sarcopenia) (*P* < 0.000). As expected, all sarcopenia criteria (i.e. muscle mass, muscle strength and gait speed) progressively decreased as the severity of the condition increased (*P* < 0.01).

**Figure 1:**
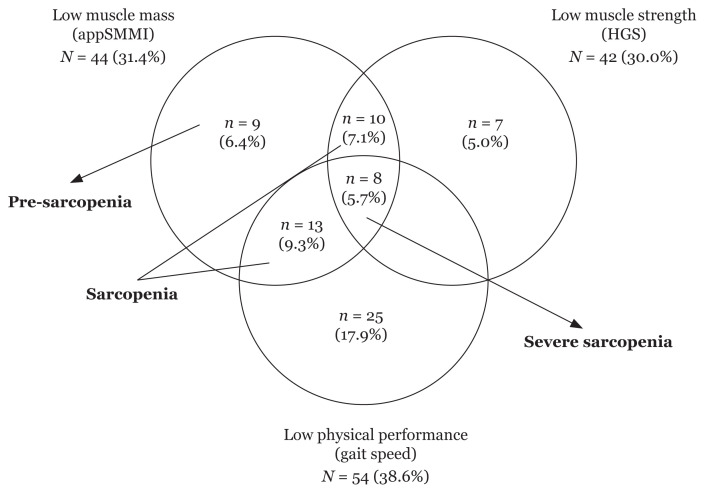
Venn diagram showing proportions of participants with low muscle mass, low muscle strength and low physical performance Notes: AppSMMI = appendicular skeletal muscle mass index, kg/m^2^; Four participants (*n* = 4) had missing values for handgrip strength (HGS) or gait speed and were not included in the categorisation for ‘sarcopenia’ and ‘severe sarcopenia’

Nearly 1/3 of the cohort had different severities of ‘sarcopenia’ (29.4% or *n* = 40/136 individuals), among which 6.4% (9/136) had ‘pre-sarcopenia’, 17% (23/136) had ‘sarcopenia’ and 5.7% (8/136) had ‘severe sarcopenia’ (Figure 1). Figure 1 shows the percentage of participants with low muscle mass, low muscle strength (dynapenia) and low gait speed (31.4%, 30.0% and 38.6%, respectively).

**Figure 2:**
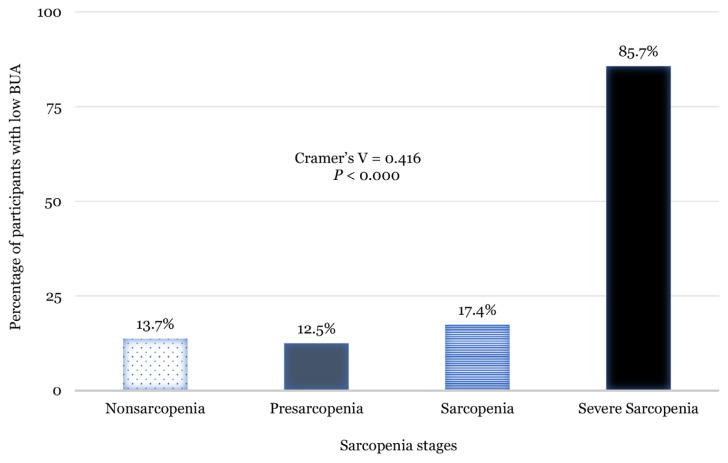
Proportion of low BUA according to each sarcopenia stage. Low BUA is defined according to Johansen et al. (50) as calcaneal BUA < 54 dB/MHz

One-way ANOVA revealed significant differences in age, adiposity (BMI, WC and BFP), muscle mass (FFMI, SMMI and appSMMI), gait speed and bone density (BUA) between the reference group and the group with ‘severe sarcopenia’ (Table 1). No significant differences were found in age, years since menopause, adiposity (BMI, WC and BFP) and whole-body muscle mass (FFMI and SMMI) across all sarcopenic groups. The results also showed no significant differences in HGS and gait speed between individuals with and without sarcopenia’ (Table 1). The rates of low BUA (i.e. low bone density) according to each stage of sarcopenia were 13.7%, 12.5%, 17.4% and 85.7% for non-sarcopenia, pre-sarcopenia, sarcopenia and severe sarcopenia groups, respectively (*P* < 0.000) (Figure 2). Pearson’s chi-squared analysis revealed significant correlations between low bone density and sarcopenia stages. All participants in each category had a BFP of ≥ 32% (obese).

To examine the extent to which age, adiposity, body weight and bone density predict the different stages of sarcopenia, multinomial logistic regression analysis was performed, with age, BFP, body weight and BUA as predictors. In this analysis, belonging to the pre-sarcopenic, sarcopenic and severe sarcopenic groups was contrasted to being associated with the non-sarcopenic group. The results showed that, without any adjustments for other variables (Table 2: crude), body weight and fat percentage were inversely correlated with each stage of sarcopenia (Table 2). However, after adjusting for BUA and age, only body weight retained the inverse correlation, while BFP became positive (Table 2: adjusted). BUA also retained its inverse relationship after adjustments, but only with ‘severe sarcopenia’. Although age had a significant and positive correlation with sarcopenia and severe sarcopenia, the significance was lost when adjusted for confounders (Table 2). The most powerful predictors of being in the severe sarcopenic group compared to the non-sarcopenic group were body weight, BFP and BUA. The best predictors of severe sarcopenia were body weight, BFP and BUA levels. The pseudo-*R*^2^ (Nagelkerke) value for the model was 0.7.

## Discussion

The present study found an almost one-third prevalence of various stages of sarcopenia in a cohort of postmenopausal Malaysian women. Results also showed that the most powerful predictors of being in a ‘severe sarcopenic’ group as opposed to being in a ‘non-sarcopenic’ group were low body weight, high BFP and low bone density (BUA). Moreover, participants with ‘sarcopenia’ had the same level of HTS and gait speed as those with severe forms of the disorder, suggesting that individuals with sarcopenia and severe sarcopenia are at the same level in terms of strength and endurance.

Studies in Western countries have found that the prevalence of sarcopenia ranges from 7.0% to 16.0% and is more likely to affect women than men. A study in the United Kingdom examining 1,787 older adults aged 60 years old or older (67 ± 2.6 years old) found that the prevalence of sarcopenia was 4.6% in men and 7.9% in women (based on the diagnosis algorithm proposed by EWGSOP). Interestingly, the indicator for low muscle mass in this study was based on the lower third of the sex-specific distribution of the fat-free mass index (FFMI) instead of appSMMI, as proposed by AWGS (23). A study from Brazil (24) which examined 1,149 older adults of a similar age group (69.6 ± 0.6 years old) living in an urban area of the municipality of São Paulo, found that the prevalence of sarcopenia in the whole population was 15.4%, with a higher prevalence in women (16.1%) compared to men (14.4%). Similar to the UK study, the prevalence in the Brazilian study was also determined by the diagnostic algorithm proposed by the EWGSOP. However, the indicator for muscle mass loss was based on appSMMI instead of FFMI, and the cut-off points were determined based on the lowest 20% of the distribution of the population (8.90 kg/m^2^ for men and 6.37 kg/m^2^ for women). Regardless, a similar comparison between studies from Asia is challenging because of differences in study design, sample size, geographical boundaries, population backgrounds, the definition of sarcopenia, diagnostic criteria, techniques and body compositions across ethnic groups (21, 25). For example, a study in Japan that examined 1,882 healthy older adult community dwellers aged between 65 years old and 89 years old (74.9 ± 5.5 years old) found that the prevalence of sarcopenia was 21.8% for men and 22.1% for women (26). The diagnostic criteria were based on the EWGSOP rather than the AWGS and SMMI was used as an indicator of muscle mass loss. Similarly, a study in South Korea that analysed 2,332 older adults aged ≥ 65 years old found that the prevalence of sarcopenia was 9.7% for men and 11.8% for women (27). Although a study in Taiwan used the same diagnostic criteria (EWGSOP), a study that assessed 2,867 individuals aged 65 years old or older (74 ± 6 years old) found that the prevalence of sarcopenia was much lower (5.4% for men and 2.5% for women). It is worth mentioning that this study used appSMMI as an indicator of muscle loss rather than whole-body muscle mass (28). These differences in muscle mass indicators and guidelines have made it almost impossible to compare findings and draw conclusions. A comparative study found that the prevalence of sarcopenia ranged from 0% to approximately 10% when different diagnostic criteria were used (International Working Group on Sarcopenia, EWGSOP or AWGS) (29). Furthermore, because ethnic differences in body composition exist, health risk cut-off points may differ between populations (30). A consensus is needed regarding the definition of sarcopenia for proper comparisons.

The current study, which used the diagnostic criteria proposed by AWGS to define sarcopenia, found that the overall prevalence of participants with various stages of sarcopenia among community-dwelling, postmenopausal Malaysian women was 29.4%, among which 6.6% had pre-sarcopenia and 5.9% had severe sarcopenia (Table 1 and Figure 1). This finding is similar to a study involving 387 community-dwelling older adults (68.3 ± 5.66 years old) in Singapore (based on diagnostic criteria proposed by AWGS), which was 27.4% (31). However, these percentages were lower than those reported by Norshafarina et al. (32) with a similar multiethnic Malaysian population (59.8%). Notably, the study by Norshafarina et al. (32) defined sarcopenia using diagnostic criteria by EWGSOP and the indicator for muscle loss was from the whole-body muscle mass index without the combination of muscle strength and/or muscle performance. Norshafarina et al. (32) also used different cut-off points, whereby the cut-points proposed by Janssen et al. (33) were used for each sarcopenia spectrum. Conversely, the current study used the cut-off points recommended by the AWGS, which used the appSMMI as an indicator to define low muscle mass in combination with either low muscle strength and/or low muscle performance. The appSMMI was chosen as an indicator of low muscle mass because approximately 75% of SMM is located in the appendicular region, which is deemed more clinically relevant (34). Evidence has shown that the reduction of appSMMI leads to negative health consequences, such as weakness, disability, impaired quality of life (QOL) and even mortality (13). Norshafarina et al. (32) also acknowledged that the prevalence of sarcopenia in their cohort was significantly higher than that documented in Western and other Asian countries.

In the current study, when analyses were performed across the severity spectrum of sarcopenia, significant differences were found between participants with and without severe sarcopenia (Table 1). Apart from having a lower appSMMI, individuals with severe sarcopenia were also found to be significantly older and skinnier, with lower gait speed and bone density than their healthier counterparts (no sarcopenia) (Table 1). Gait speed is a crucial factor in determining neuromuscular quality and has been used to diagnose functional disability and dependence in older adults (11). Loss of independence in activities of daily living (ADL) is closely correlated with sarcopenia and slows gait speed. Therefore, gait speed should be improved through physical training and exercise. Published findings have shown that the deleterious effects of sarcopenia may be mitigated by a better gait speed profile (35). Furthermore, the current study also found that individuals with ‘sarcopenia’ had the same level of HGS and gait speed as those with severe forms of the disorder (no significant difference), implying that individuals with sarcopenia and severe sarcopenia are at the same level in terms of strength and endurance (Table 1). This finding is concerning because the risk of frailty is likely imminent with only a diagnosis of ‘sarcopenia’. Sarcopenia and frailty are severely detrimental to older adults, including a higher risk of falls and fractures, decreased ability to perform daily tasks, declining cognitive function, loss of independence, the requirement for long-term care and death (36). Frailty and sarcopenia are interlinked processes that can be triggered or worsened by a sudden illness or injury. Therefore, a periodic evaluation is required to mitigate the impact of these two conditions. It is well established that muscle mass and strength decrease with ageing, although more prominent after the age of 40 years old (37, 38). Loss of muscle mass in conjunction with weight loss in older adults may cause serious functional disabilities and physical limitations in their daily lives.

As people age, their amount of fat tissue increases, while their percentage of skeletal muscle declines owing to irregularities in the hormones that regulate their energy metabolism (39–41). According to the findings of the current study, the higher the BFP, the higher the likelihood of severe sarcopenia (Table 2; adjusted). Conversely, severe sarcopenia was associated with low bone density (Tables 1 and 2, and Figure 2). The simultaneous presence of low muscle mass (sarcopenia), low bone density (osteoporosis), and high adiposity (obesity), otherwise known as OSO can be detrimental in older adults (14).

OSO is an age-related disorder that is considered the most advanced functional impairment related to bone, muscle and adiposity (18). Studies have shown that low oestrogen levels and low vitamin D, and a sedentary lifestyle are some of the most significant risk factors shared by all three disorders (42). Therefore, the current hypothesis is that women who reach menopause would have a higher risk of developing OSO due to their sudden drop in oestrogen level, age-related increase in adiposity and low physical activity. Individuals presenting all three conditions concurrently are expected to have poorer clinical outcomes than individuals with either condition alone. A study in China showed that older women with OSO had a lower quality blood lipid profile (43) than those without OSO, while a Mexican study found that women with OSO had significantly lower physical performance and poorer frailty scores than those without the syndrome (44). Furthermore, Ilich et al. (45) found a strong correlation between OSO and weaker HGS and inferior balance and walking abilities in older women compared to obese-only women of similar age. Metabolic abnormalities, such as dyslipidaemia and insulin resistance (IR), were also found to be worse when compared to the co-existence of all three disorders (46). Therefore, owing to the close link between muscle, bone and fat, further studies are needed on OSO, particularly in terms of diagnosis and treatment.

The current study also supports previous findings suggesting a link between sarcopenia and osteoporosis in postmenopausal women (47). The current study found that the rate of low bone density increased with sarcopenia severity (Figure 2). Based on these findings, severe sarcopenia, defined as reduced muscle mass, strength and function, increases the risk of low bone density. Postmenopausal women with severe sarcopenia may be at a higher risk for fractures and related complications because, in addition to having more fragile bones, they are also more prone to falls and, therefore, should be monitored by health professionals involved in bone health promotion. Sarcopenia and osteoporosis are musculoskeletal diseases that are expected to become more common as the global population ages. Both disorders have similar etiological processes, and evidence suggests that sarcopenia and osteoporosis are closely related.

The key findings suggest that low muscle mass, high adiposity and low bone density are interlinked. OSO is a syndrome that progresses and requires early treatment. The risk of adverse health outcomes in old age may be decreased in asymptomatic obese women through screening for osteosarcopenia. The development of behavioural, nutritional and even pharmacological interventions to prevent or treat OSO will eventually increase scientific and public awareness of the proper diagnostic criteria, prognosis and public health costs due to the recognition of OSO as an emerging public health problem.

## Conclusion

In summary, the present study documented a high prevalence of sarcopenia (29.4%) among Malaysian postmenopausal women compared to published reports from other Asian countries. Severe sarcopenia is associated with low bone density and high adiposity. Future studies should focus on establishing OSO as a single entity and identifying methods to manage and treat the syndrome. Patients with normal and severe sarcopenia suffered some serious impairments. Therefore, early intervention is vital to stop the progression from pre-sarcopenia to confirmed sarcopenia.

### Limitations and Strengths

This study has some limitations. The participants in this study were primarily healthy and relatively young (~50 years old), the age at which muscle mass loss begins to progress. This may have resulted in a lower prevalence of sarcopenia, thus limiting the extrapolation of the results. The quantitative ultrasound (QUS) was used to measure bone density. The advantages of QUS are its ease of use and non-invasive method of estimating bone mineral status in the peripheral skeleton. The QUS technique is radiation-free, safe and economical. The instruments are portable and measurements can be completed in a short amount of time. However, QUS was not recommended as a means of diagnosis. Instead, individuals with low QUS results should be referred for bone mineral density (BMD) assessment using gold standard dual-energy X-ray absorptiometry (DXA). Nevertheless, studies have shown a good correlation between QUS and DXA (48). Dane et al. (49) reported that all three QUS indices, BUA, SOS and SI, were significantly correlated with BMD assessed by DXA of the lumbar spine and femur in postmenopausal women. While ultrasound parameters do not directly measure BMD, BUA and SOS results have been found to correlate (*R* = 0.82–0.85) with heel BMD results obtained using the standard DXA technique, as are the results for the combined QUI parameter (*R* = 0.85). Future studies should be conducted using the gold standard DXA to validate these results. Notwithstanding these limitations, the current study had several strengths. One of the strengths of this study lies in the findings of the correlations between low muscle mass, low bone density and high adiposity. The current study also adds to the expanding body of evidence showing that sarcopenia and low bone density frequently occur together and provides the unexpected insight that bone health deteriorates as sarcopenia progresses. However, to achieve clinical significance, the literature must be understood in light of its practical application. Incorporating the current understanding into the prevention, screening and treatment of obesity, sarcopenia and osteoporosis would be highly beneficial.

## Figures and Tables

**Table 1:** Descriptive characteristics of the participants stratified according to each stage of sarcopenia and its between-group comparisons. Data are expressed as mean and standard deviation

Variables	*N*	No sarcopenia, *n* = 96 mean (SD)	Pre-sarcopenia, *n* = 9 mean (SD)	Sarcopenia, *n* = 23 mean (SD)	Severe sarcopenia, *n* = 8 mean (SD)	*P*-value	Eta^2^
Age (years old)	136	59.1 (6.7)ƚ	63.0 (9.0)	62.84 (7.4)	65.88 (11.0)	**0.010** *	**0.083**
Years since menopause (years)	115	8.6 (6.5)	9.8 (6.6)	12.7 (8.7)	13.50 (11.2)	0.077*	0.060
Height (cm)	136	154.1 (5.8)ƚ	151.1 (6.7)	152.0 (6.8)	147.8 (6.17)	**0.017** *	**0.074**
Weight (kg)	136	68.9 (10.5)δ, β, ƚ	50.0 (8.2)	53.4 (6.4)	47.16 (8.48)	**0.000** *	**0.417**
Body mass index (kg/m^2^)	136	29.1 (4.8)δ, β, ƚ	21.8 (2.8)	23.3 (3.6)	21.63 (4.22)	**0.000** *	**0.311**
WC (cm)	133	88.6 (11.3)δ, β, ƚ	73.8 (13.0)	75.3 (8.7)	72.86 (8.54)	**0.000** *	**0.273**
Body fat (%)	136	42.4 (7.2)δ, β, ƚ	34.3 (9.1)	37.9 (7.4)	35.35 (7.9)	**0.000** *	**0.129**
FFMI (kg/m^2^)	136	16.5 (1.3)δ, β, ƚ	14.1 (0.7)	14.2 (1.0)	13.70 (1.14)	**0.000** *	**0.451**
SMMI (kg/m^2^)	136	8.9 (0.8)δ, β, ƚ	7.4 (0.4)	7.4 (0.5)	7.05 (0.6)	**0.000** *	**0.481**
AppSMMI (kg/m^2^)	136	6.5 (0.7)δ, β, ƚ	5.3 (0.2) ƚ	5.3 (0.4)ƚ	4.8 (0.58)	**0.000** *	**0.515**
Handgrip strength (kg)	129	20.7 (5.0)ƚ	22.6 (3.4)ƚ	17.9 (4.4)	14.9 (1.5)	**0.001** *	**0.131**
Gait speed (m/s)	127	0.9 (0.3)ƚ	1.1 (0.1)ƚ	0.8 (0.2)	0.7 (0.1)	**0.005** *	**0.099**
BUA (dB/MHz)	134	72.2 (16.0)ƚ	73.7 (16.4)ƚ	65.9 (15.9)ƚ	47.9 (8.86)	**0.001** *	**0.114**

Notes: SD = standard deviation; Pre-sarcopenia = low muscle mass without impact on muscle strength or physical performance; sarcopenia = low muscle mass, plus low muscle strength or low physical performance; severe sarcopenia = low muscle mass, plus low muscle strength and low physical performance; BUA = broadband ultrasonic attenuation; WC = waist circumference; SMMI = skeletal muscle mass index; FFMI = fat-free mass index; AppSMMI = appendicular skeletal muscle mass index;

* analysed using one-way ANOVA with Tukey’s HSD post-hoc test, *P* < 0.05;

β different from sarcopenia *P* < 0.05;

δ different from pre-sarcopenia;

ƚ different from severe sarcopenia *P* < 0.05;

value in bold is significant

**Table 2:** Summary of multinomial logistic regression analysis for multiple variables predicting membership in groups of pre-sarcopenia, sarcopenia and severe sarcopenia

Group^a^	Variable	*β*	Std. error	Wald^b^	*P*-value	OR	95% CI (lower bound–upper bound)
**Crude**							
Pre-sarcopenia	Constant	−2.887	1.674	2.976	0.085		
	BUA (dB/MHz)	0.006	0.022	0.062	0.804	1.006	0.963, 1.050
	Constant	−6.763	2.869	5.557	0.018		
	Age (years old)	0.072	0.046	2.506	0.113	1.075	0.983, 1.175
	Constant	15.000	3.740	16.083	0.000		
	Weight (kg)	−0.300	0.070	18.517	**0.000**	0.741	0.647, 0.849
	Constant	2.925	1.688	3.003	0.083		
	BFP (%)	−0.137	0.046	8.793	**0.003**	0.872	0.796, 0.955
Sarcopenia	Constant	0.241	1.076	0.050	0.823		
	BUA (dB/MHz)	−0.024	0.016	2.400	0.121	0.976	0.947, 1.006
	Constant	−5.955	1.975	9.091	0.003		
	Age (years old)	0.074	0.032	5.511	**0.019**	1.077	1.012, 1.146
	Constant	11.985	2.644	20.555	0.000		
	Weight (kg)	−0.223	0.046	23.541	**0.000**	0.800	0.731, 0.875
	Constant	1.822	1.261	2.086	0.149		
	BFP (%)	−0.081	0.032	6.499	**0.011**	0.923	0.867, 0.982
Severe sarcopenia	Constant	6.867	2.626	6.840	0.009		
	BUA (dB/MHz)	−0.165	0.052	10.206	**0.001**	0.848	0.766, 0.938
	Constant	−9.577	3.026	10.017	0.002		
	Age (years old)	0.114	0.046	6.052	**0.014**	1.121	1.023, 1.227
	Constant	17.899	4.085	19.200	0.000		
	Weight (kg)	−0.362	0.079	21.124	**0.000**	0.696	0.597, 0.813
	Constant	2.251	1.783	1.594	0.207		
	BFP (%)	−0.121	0.048	6.393	**0.011**	0.886	0.807, 0.973
**Adjusted**							
Pre-sarcopenia	Constant	25.505	9.154	7.763	0.005		
	BUA (dB/MHz)	0.018	0.027	0.442	0.506	1.018	0.966, 1.073
	Age (years old)	−0.085	0.081	1.098	0.295	0.918	0.783, 1.077
	Weight (kg)	−0.638	0.156	16.745	**0.000**	0.528	0.389, 0.717
	BFP (%)	0.330	0.113	8.500	**0.004**	1.391	1.114, 1.736
Sarcopenia	Constant	17.734	6.195	8.193	0.004		
	BUA (dB/MHz)	−0.014	0.020	0.515	0.473	0.986	0.948, 1.025
	Age (years old)	−0.013	0.054	0.058	0.810	0.987	0.888, 1.097
	Weight (kg)	−0.494	0.108	20.786	**0.000**	0.610	0.493, 0.754
	BFP (%)	0.305	0.087	12.300	**0.000**	1.357	1.144, 1.610
Severe sarcopenia	Constant	27.115	8.863	9.360	0.002		
	BUA (dB/MHz)	−0.124	0.053	5.519	**0.019**	0.884	0.797, 0.980
	Age (years old)	−0.010	0.077	0.018	0.893	0.990	0.851, 1.150
	Weight (kg)	−0.642	0.172	13.889	**0.000**	0.526	0.375, 0.738
	BFP (%)	0.383	0.151	6.466	**0.011**	1.467	1.092, 1.971

Notes: *N* = 136; BUA = broadband ultrasonic attenuation; BFP = body fat percent; *β* = estimated coefficient for the predictor; OR = odds ratio; CI = confidence interval;

a The reference group is No Sarcopenia;

b df = 1; value in bold is significant
